# Whole-Genome Sequences of Three *Streptococcus macedonicus* Strains Isolated from Italian Cheeses in the Veneto Region

**DOI:** 10.1128/genomeA.01358-17

**Published:** 2017-12-14

**Authors:** Laura Treu, Beatriz de Diego-Díaz, Konstantinos Papadimitriou, Effie Tsakalidou, Alessio Giacomini, Viviana Corich

**Affiliations:** a Department of Environmental Engineering, Technical University of Denmark, Kongens Lyngby, Denmark; b Department of Chemistry, University of Navarra, Navarra, Spain; c Department of Food Science and Human Nutrition, Agricultural University of Athens, Athens, Greece; d Department of Agronomy, Food, Natural Resources, Animals and Environment (DAFNAE), University of Padua, Padua, Italy

## Abstract

We report here the genome sequences of three *Streptococcus macedonicus* strains isolated from different cheeses in the Veneto region of Italy. The presented data aim at increasing the scarce genomic information available for this species, which is frequently encountered in fermented foods and appears to be a promising technological microorganism.

## GENOME ANNOUNCEMENT

*Streptococcus macedonicus* is a bacterial species quite frequently recovered from fermented milks and cheeses (1, 2). The study of its properties suggests a potential interesting technological role in determining characteristics of cheese (3). However, the possible presence of pathogenic traits must be fully elucidated in order to classify *S. macedonicus* under the Qualified Presumption of Safety (QPS, in the European Union) or Generally Recognized as Safe (GRAS, in the United States) status.

The present work doubles the number of genomes available for this species since, to date, only three sequenced genomes are publicly available. The sequenced strains were isolated from cheeses produced in the Veneto region of Italy. In detail, strain 19AS comes from Asiago cheese, strain 27MV from Monte Veronese cheese, and strain 211MA from a “malga” (pasture) cheese.

The genome sequencing was performed with an Illumina MiSeq sequencer and Nextera XT libraries at the Ramaciotti Centre, Sydney, Australia. The average number of paired-end reads (2 × 250 bp) for the three strains was 1,991,895, with coverages of 223-fold, 216-fold, and 251-fold, for strains 19AS, 27MV, and 211MA, respectively. The total number of scaffolds obtained was between 38 and 102, and the obtained genomes for strains 19AS, 27MV, and 211MA have total sizes of 2.3, 2.3, and 2.0 Mb, respectively, with a GC content of 37.3%. Read filtering, pair merging, and assembly were completed using CLC Genomics Workbench version 10.1.1 (QIAGEN Bioinformatics) with standard parameters and automatic bubble size. The scaffolds of all the strains were combined into a circular chromosome through alignment with Mauve software version 2.3.1 against a reference genome, i.e., *S. macedonicus* ACA-DC198.

The Rapid Annotations using Subsystems Technology (RAST) server was used for gene prediction and annotation (4). The numbers of predicted protein-coding sequences (CDSs) in 19AS, 27MV, and 211MA were, respectively, 2,360, 2,419, and 2,108, distributed on an average of 321 subsystems. Furthermore, 46, 45, and 45 RNA genes were found for strains 19AS, 27MV, and 211MA, respectively. Strain 211MA does not contain phage sequences or transposase-encoding genes, while 19AS and 27MV have 16 and 38, respectively. Additionally, 3 to 4 clusters of regularly interspaced short palindromic repeats (CRISPRs) are present in all the strains.

From the comparison with *S. macedonicus* genomes publicly available, i.e., ACA-DC198 (2,160 CDSs) (5), 679 (2,296 CDSs) (6), and 33MO (2,179 CDSs) (7), strains 19AS and 27 MV contain a greater number of CDSs. Additionally, all strains contain bacteriocin-related sequences, 6 each for 27MV and 211MA and 10 for 19AS, thus confirming reported findings on the ability of *S. macedonicus* strains to produce antimicrobial peptides (8). With respect to sequenced *S. thermophilus* strains (9–11), *S. macedonicus* strains possess up to 10 genes related to bacteriocins and ribosomally synthesized antimicrobial peptides. This is a larger number with respect to those reported for eight strains of *S. thermophilus* (12). Also, the three strains contain a mean count of 232 features for amino acid metabolism, from which an average number of 39 are linked to the metabolism of arginine, a key molecule in several pathways of lactic acid bacteria involved in food production (13).

### Accession number(s).

This whole-genome shotgun project has been deposited at DDBJ/ENA/GenBank under the accession numbers PEBN00000000, PEBM00000000, and PEBL00000000 for *S. macedonicus* strains 19AS, 27MV, and 211MA, respectively. The versions described in this paper are the first versions, PEBN01000000, PEBM01000000, and PEBL01000000, respectively.
